# An *in vivo *mouse model of long-term potentiation at synapses between primary afferent C-fibers and spinal dorsal horn neurons: essential role of EphB1 receptor

**DOI:** 10.1186/1744-8069-5-29

**Published:** 2009-06-12

**Authors:** Wen-Tao Liu, Yuan Han, Hao-Chuan Li, Brandt Adams, Ji-Hong Zheng, Yong-Ping Wu, Mark Henkemeyer, Xue-Jun Song

**Affiliations:** 1 Jiangsu Province Key Laboratory of Anesthesiology and Center for Pain Research and Treatment, Xuzhou Medical College, Xuzhou, Jiangsu 221002, PR China; 2 Department of Neurobiology, Parker University Research Institute, 2500 Walnut Hill Lane, Dallas, Texas 75229, USA; 3 Department of Developmental Biology, University of Texas Southwestern Medical Center, Dallas, Texas 75390, USA; 4 Department of Anesthesiology, Xijing Hospital, The Fourth Military Medical University, Xi'an, Shanxi 710032, PR China

## Abstract

**Background:**

Long-term potentiation (LTP), a much studied cellular model of synaptic plasticity, has not been demonstrated at synapses between primary afferent C-fibers and spinal dorsal horn (DH) neurons in mice *in vivo*. EphrinB-EphB receptor signaling plays important roles in synaptic connection and plasticity in the nervous system, but its role in spinal synaptic plasticity remains unclear.

**Results:**

This study characterizes properties of LTP at synapses of C-fibers onto neurons in the superficial DH following high-frequency stimulation (HFS) of a peripheral nerve at an intensity that activates C-fibers and examines associated activation of Ca^2+^/calmodulin-activated protein kinase II (p-CaMKII), extracellular signal-regulated kinase (p-ERK) and the cyclic AMP response element binding protein (p-CREB) and expression of c-Fos, and it investigates further roles for the EphB1 receptor in LTP. HFS induced LTP within 5 min and lasts for 3–8 h during the period of recording and resulted in upregulation of p-CaMKII, p-ERK and p-CREB protein levels in the spinal cord and expression of c-Fos in DH. Intrathecal pretreatment of MK-801 or EphB2-Fc prevented LTP and significantly reduced upregulation of p-CaMKII, p-ERK, p-CREB and c-Fos. Further, targeted mutation of EphB1 receptor prevented induction of LTP and associated increases in phosphorylation of CaMKII, ERK, and CREB.

**Conclusion:**

This study provides an *in vivo *mouse model of LTP at synapses of C-fibers onto the superficial DH neurons that will be valuable for studying the DH neuron excitability and their synaptic plasticity and hyperalgesia. It further takes advantage of examining functional implications of a specific gene targeted mice and demonstrates that the EphB1 receptor is essential for development of LTP.

## Background

Long-term potentiation (LTP) is a much studied cellular model of synaptic plasticity and it is often studied using *in vitro *preparations. LTP at synapses in the hippocampus is the predominant model for learning and memory formation [1,2]. *In vivo *studies using rats have shown that LTP can also be induced in pain pathways at synapses between primary afferent C-fibers and dorsal horn (DH) neurons of the spinal cord (SC) [3-5]. This LTP is thought to be associated with sensitization of the DH neurons and contributes to hyperalgesia caused by inflammation, trauma or neuropathy [3,6-9]. While gene targeted mutant mice have been used to advantage in biomedical studies for decades, an in vivo model for LTP in the DH of this species has not been established. The development of such a new model would be very valuable for studying sensitization of the DH neurons and their synaptic plasticity and behavioral hyperalgesia. We characterize here the LTP of synapses between primary afferent C-fibers and superficial DH neurons in intact, anesthetized mice *in viv*o.

LTP can be induced and/or expressed by presynaptic [10] and postsynaptic [11,12] mechanisms. Although LTP has been studied extensively in various synapses, mechanisms of LTP ay synapses between the C-fibers and the DH neurons are still poorly understood. Recent studies have provided evidence for a postsynaptic, NMDA mediated, Ca^2+^-dependent form of LTP induction in lamina I neurons of rat SC [8,13]. The Ca^2+^-dependent pathways include protein kinase C (PKC), calcium-calmodulin-activated protein kinase II (CaMKII), protein kinase A (PKA), nitric oxide synthase (NOS) and members of the mitogen-activated protein-kinase family (MAPK), including the extracellular signal-regulated kinase (ERK) [6,8,13-15]. We here provide our *in vivo *evidence in mice that high frequency stimulation (HFS) at C-fiber intensity of sciatic nerve afferents produces LTP associated with phosphorylation of CaMKII (p-CaMKII), ERK (p-ERK) and CREB (p-CREB), and increased expression of c-Fos in the SC.

We have recently reported that ephrinB-EphB receptor signaling, which is important in synaptic plasticity in the nervous system and regulates the glutamatergic synapses and their plasticity by interaction with NMDA receptors [16-26], contributes to LTP in intact, anesthetized rats *in viv*o [9]. Because of the lack of reagents and antibodies that selectively activate and/or block specific members of the EphB receptor subclasses, the identity of the EphB receptor that may be involved in LTP has not been identified. Here, using this new *in vivo *model of LTP in mice, we analyzed animals that were homozygous mutant for the EphB1 receptor (*EphB1*^-/-^) and provide evidence that this receptor is essential to development of LTP. Our analysis further suggests the EphB1 receptor mediates LTP by modulating the previously characterized Ca^2+^-dependent pathways.

## Results

### Properties of C-fibers-evoked field potentials in the mouse DH in vivo

**Figure 1:**
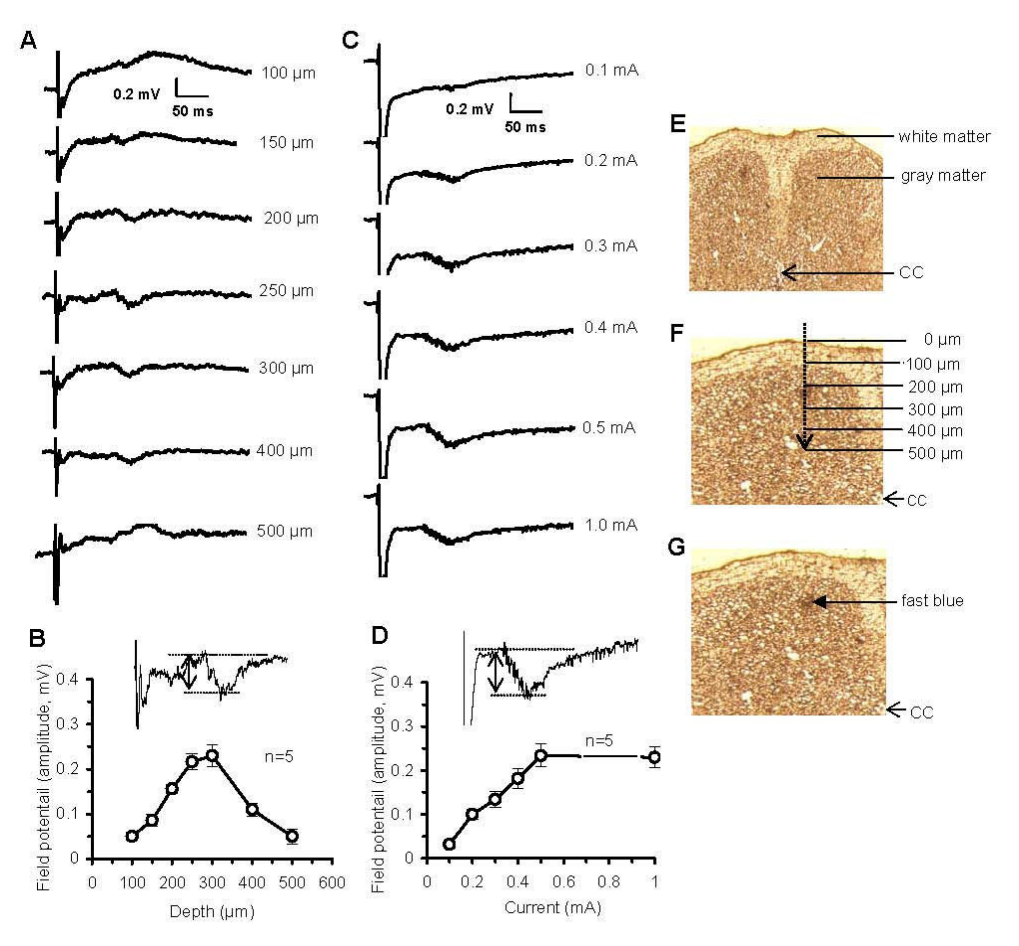
**Primary afferent C-fibers evoked field potentials in the mouse DH *in vivo***. A, B: Examples and data summary of the field potentials evoked by electrical stimulation (0.5 mA, 0.5 ms) of the sciatic nerve at different laminae of DH (100–500 μm from the surface of SC, also see F). C, D: Examples and data summary of the field potentials evoked by electrical stimulation (0.5 ms, with different strength) of the sciatic nerve in the same site of DH (~220 μm from the surface of SC, also see G). E-G: Histological verification of the recording site in DH. The tissues were sectioned freshly and examined under microscope. The images F and G were copied and edited from the left side of DH in E. CC: central canal.

The C-fibers-evoked field potentials characterized by prominent negative components are a reflection of the extracellularly-recorded excitation of DH neurons by stimulated the primary C-fiber afferents. Changes in the field potential indicate alterations in the synaptic activity of the DH neurons. After supramaximal electrical stimulation (0.5–1.0 mA, 0.5 ms) of the sciatic nerve, the field potential was recorded in the mouse DH (100–500 μm from the surface of SC). The field potential displayed a negative peak at relatively long latency (mean ± SE, 71.1 ± 2.9 ms, ranged 60–95 ms, corresponding to conduction velocities 0.52 ± 0.01 m/s; the distance from the stimulation site at the sciatic nerve to the recording site 3.6 ± 0.1 cm, n = 15), and a high threshold during nerve stimulation (≥ 0.2 mA, 0.5 ms). The field potential could be recorded at depth of 100–500 μm but the maximal potentials were at approximately 200–300 μm (Fig. 1A, B). Stimulation of the sciatic nerve at strength of 0.5–1.0 mA (0.5 ms) activated approximately all the C-fibers and the maximum field potential was recorded (Fig. 1C, D). All of the field potentials were recorded at 1-min intervals and 5 recordings were averaged and presented as 1 datum point (Fig. 1B, D). The depth was determined by the digital micromanipulator and was verified histologically in some samples (Fig. 1E–G). The field potentials recorded for 3 h (n = 5) or 6 h (n = 3) was stable in the amplitudes and latencies (data not shown).

To exclude the possibility that long latencies of the field potentials are due to activation of supraspinal loop or muscle contraction, in 3 experiments the stable field potential was recorded for 30–40 min and then 0.1 ml lidocaine (2%) was injected into the SC at C3–4 segment followed by surgical transaction of SC at the same site. After spinalization, latencies of the field potentials remained constant but their amplitudes increased to 140–210% of control. These properties are similar to those recorded in the rat DH [4].

### Properties of LTP in the mouse DH in vivo

**Figure 2:**
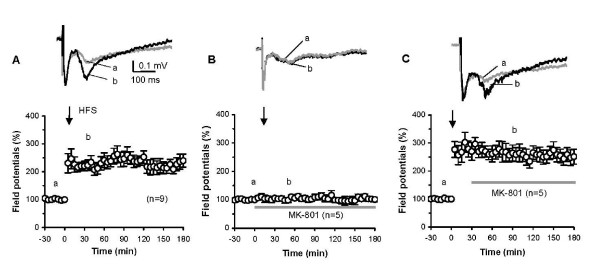
**LTP of synapses between C-fibers and DH neurons in mice *in vivo***. LTP training protocol in A-C: 100 Hz, 5 × threshold currents, 0.5 ms, 100 pulses, 4 trains of 1-s duration at 10-s intervals. Examples of the C-fiber-evoked field potentials given in each figure were recorded before (a) and after (b) tetanic stimulation (indicated by the arrow in each figure) alone (A) or combined with MK-801 with pre-treatment (B) and post-treatment (C), respectively. The nonparametric Wilcoxon signed-rank test and the Kruskal-Wallis test were used to test the field potential within and between the groups, respectively (*p *< 0.01 in A and C; *p *> 0.05 *in B*; *p *< 0.01 between A/B and B/C; *p *> 0.05 between A/C).

The C-fibers-evoked field potential was evoked in the mouse DH by stimulation of the sciatic nerve with single test pulses (0.5 mA, 0.5 ms, given at 1-min intervals). Stable responses for 30 min served as controls. A conditioning HFS was then delivered to the sciatic nerve followed by single test pulses with stimulation parameters identical to controls. The high-intensity HFS induced LTP of the field potential in 8 of 9 mice tested, while failed to induce LTP in one of the mice. Significant enhancement of amplitudes of the field potentials (range: 150–330% of baseline) lasted until the end of the recording periods (3 h in 6 mice and 8 h in 2 mice). Mean amplitudes and time course of the LTP in these experiments are illustrated in Fig. 2A. These properties of the LTP in the mouse DH are similar to those recorded in the DH of rats [4,9]. Further, the LTP was completely prevented by spinal pre-treatment with an NMDA receptor antagonist MK-801 (5 μg, n = 5, 30 min prior to, during and up to 3 h after HFS) (Fig. 2B), but not interrupted by post-treatment of MK-801 (5 μg, n = 5, 0.5–3 h after HFS) (Fig. 2C). These results confirm that LTP in the mouse DH is NMDA receptor-dependent.

### Activation of CaMKII, ERK and CREB proteins and expression of c-Fos in the mouse SC following HFS

**Figure 3:**
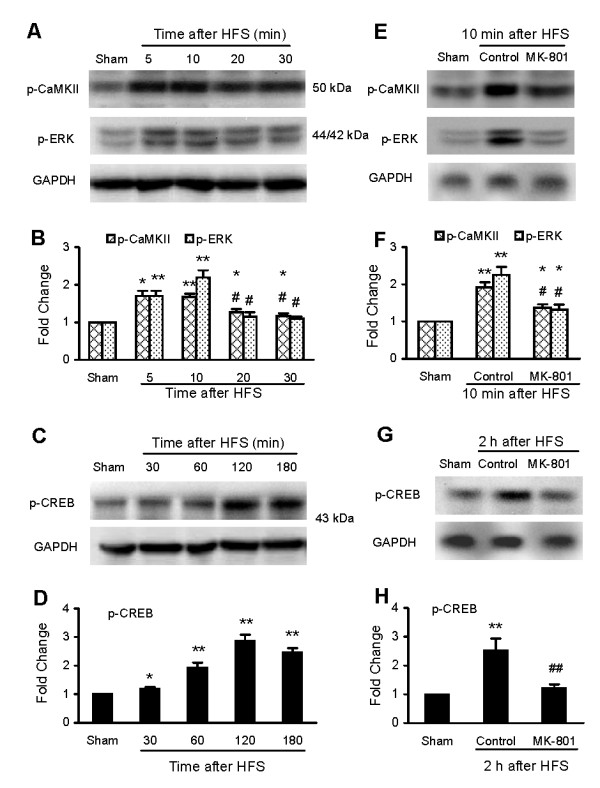
**Immunoblot analysis of phosphorylation of CaMKII, ERK and CREB proteins in the mouse SC following HFS *in vivo***. A-D: Representative immunoblots illustrating time courses of changes in CaMKII (A), pERK (A) and pCREB (C) and quantification of CaMKII (B), pERK (B) and pCREB (D) protein levels following HFS. E-H: Representative immunoblots illustrating changes of expression of the CaMKII (E), pERK (E) and pCREB (G) and quantification of CaMKII (F), pERK (F) and pCREB (H) protein levels with or without MK-801 treatment. Fold changes are standardized by protein level in the corresponding group of "sham" (surgery without HFS, mean value set as 1). *p < 0.05, **p < 0.01 indicates significant differences compared with that in the corresponding sham group; #*p *< 0.05, ##*p *< 0.01 indicates significant differences compared with that in the corresponding group of peak value 5 or 10 min after HFS (in B) and of "control" (surgery plus HFS) in F and H.

Upregulation of p-CaMKII, p-ERK, p-CREB and c-Fos expression has been used as indicators of neural activity and plasticity. We used phosphor-specific antibodies in immunoblots [27] detect the activated forms of these molecules following HFS in the SC. The results showed that HFS significantly increased levels of p-CaMKII, p-ERK, and p-CREB protein. As shown in Fig. 3A–D, the levels of p-CaMKII and p-ERK quickly increased within 5 min, the first data point after HFS, and reached their peak values in 5–10 min. The signal for p-ERK quickly recovered to near baseline levels within 20 min. The signal for p-CaMKII also decreased 20–30 min after HFS, but its level remained was still significantly higher than sham control (Fig. 3A, B). In contrast, the level of p-CREB protein increased with more delayed dynamics and reached peak level in 2–3 h post HFS (Fig. 3C, D). Our results further showed that treatment with the NMDA receptor antagonist MK-801 (5 μg, 30 min prior to, during and 10 min after HFS) significantly reduced the level of p-CaMKII and p-ERK at 10 min (Fig. 3E, F) and p-CREB at 2 h (Fig. 3G, H) after the HFS.

**Figure 4:**
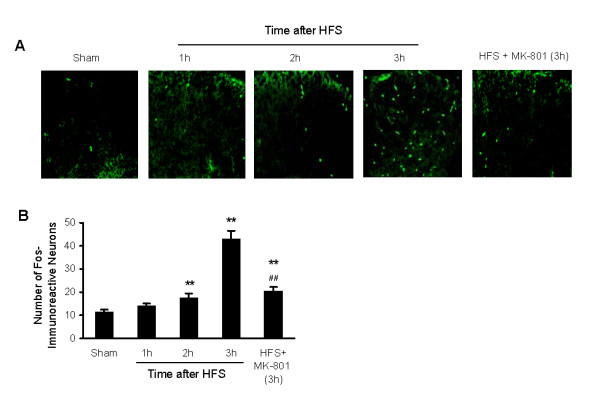
**Time course of c-Fos expression in the mouse DH after HFS *in vivo***. Examples of Fos-immunoreactive neurons are shown in A and data summarized in B. Five groups of mice (n = 5 each group) were tested and examined, the sham group received no HFS and the others received HFS without or with MK-801 treatment. ** *p *< 0.01 indicates significant differences compared with the sham. ## *p *< 0.01 indicate significant difference compared with the time point at 3 h after HFS.

We also used immunohistochemical and immunofluorescence staining to measure expression of c-Fos in the DH following HFS *in vivo*. Representative photomicrographs and the corresponding counts of Fos-like immunoreactive neurons in the DH are shown in Fig. 4. The number of Fos-immunoreactive neurons was significantly increased at 2 h and 3 h by approximately 170% and 420%, respectively, but not at 1 h after the HFS. Fos expression was not significantly changed at 1 h after HFS. Increased expression of c-Fos was markedly reduced by treatment with MK-801 (5 μg, covering from 30 min prior to, during and 3 h after HFS).

### LTP in the mouse DH in vivo was prevented by either EphB2-Fc or targeted mutation of EphB1 receptor

**Figure 5:**
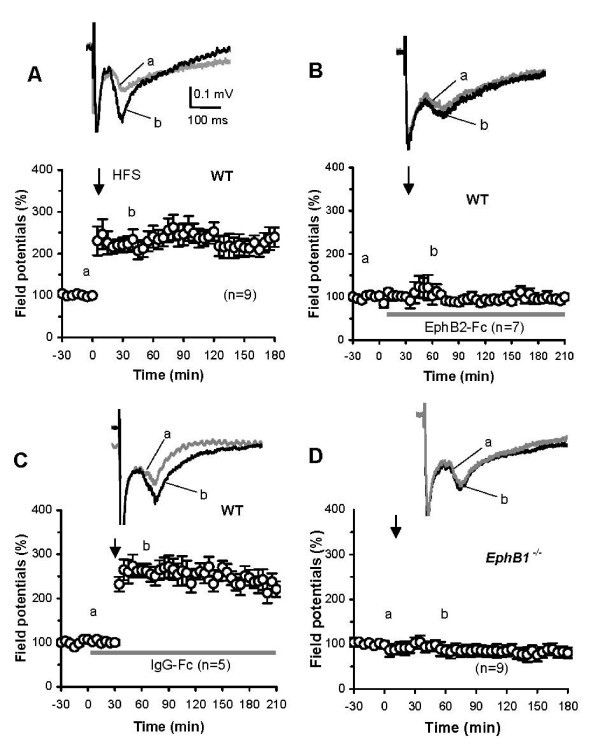
**Effects of EphB2-Fc and targeted mutation of EphB1 receptor on the LTP of synapses between C-fibers and DH neurons in mice *in vivo***. LTP training protocol in A-D was the same as that in Fig. 2A–C: 100 Hz, 5 × threshold currents, 0.5 ms, 100 pulses, 4 trains of 1-s duration at 10-s intervals. Examples of the C-fiber-evoked field potentials given in each figure were recorded before (a) and after (b) tetanic stimulation (indicated by the arrow in each figure) alone (A, the same figures and data used as control as those in Fig. 2A) or combined with pre-treatment of EphB2-Fc (B) and IgG-Fc (C), respectively. The nonparametric Wilcoxon signed-rank test and the Kruskal-Wallis test were used to test thefield potentials within and between the groups, respectively (*p *< 0.01 in A and C; *p *> 0.05 in B and D; *p *< 0.01 between A/B, B/C, A/D and C/D; *p *> 0.05 between A/C and B/D).

We investigated the role of the EphB1 receptor in the LTP by first confirming that an EphB receptor blocker that inhibits LTP in rats [9] also does so in mice. The results showed that LTP induced in WT mice (Fig. 5A) was successfully prevented by pre-application of a soluble EphB receptor blocker EphB2-Fc (2 μg, 30 min prior to, during and up to 3 h after HFS) (Fig. 3B), but not by post-treatment of the EphB2-Fc (data not shown). EphB2-Fc did not alter baselines of the C-fibers-evoked field potentials. The IgG-Fc (Fc control) did not alter the LTP amplitude (Fig. 5C). Further, our results showed that targeted mutation of the EphB1 receptor completely abolished the LTP in all mice tested (n = 9) (Fig. 5D). These genetic data strongly suggest that this EphB1 receptor is the specific Eph family receptor critical to and required for development of LTP.

### Activation of CaMKII, ERK and CREB proteins and expression of c-Fos following HFS were prevented by either EphB2-Fc or targeted mutation of the EphB1 receptor

**Figure 6:**
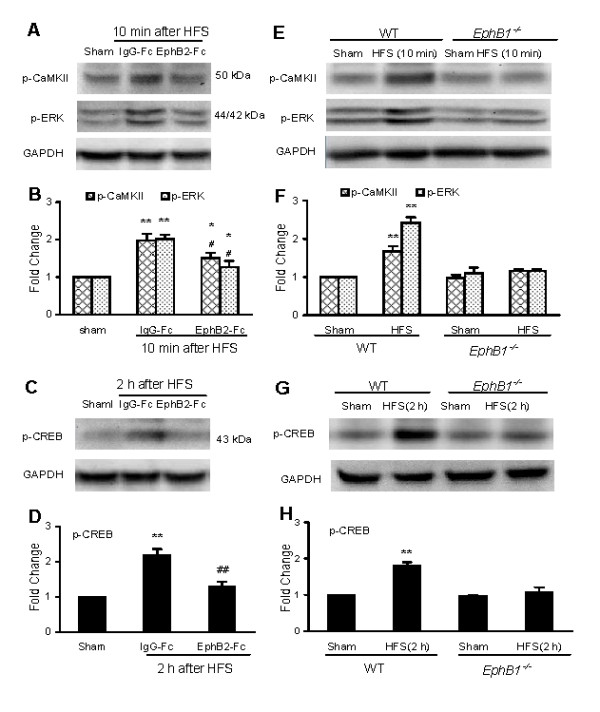
**Effects of EphB2-Fc and targeted mutation of EphB1 receptor on phosphorylation of CaMKII, ERK and CREB proteins in the mouse SC following HFS**. A-D: Representative immunoblots illustrating changes in HFS-induced phosphorylation of CaMKII (A), pERK (A) and pCREB (C) and quantification of CaMKII (B), pERK (B) and pCREB (D) proteins after pretreatment of EphB2-Fc. E-H: Representative immunoblots illustrating changes in HFS-induced phosphorylation of CaMKII (E), pERK (E) and pCREB (G) and quantification of CaMKII (F), pERK (F) and pCREB (H) proteins in wild type (WT) and EphB1 receptor protein-null (*EphB1*^-/-^) mice. Fold changes are standardized by protein level in the corresponding group of "sham" (surgery without HFS, mean value set as 1). *p < 0.05, **p < 0.01 indicates significant differences compared with that in the corresponding sham group; #*p *< 0.05, ##*p *< 0.01 indicates significant differences compared with that in the corresponding group of IgG-Fc (as control) in B and D.

**Figure 7:**
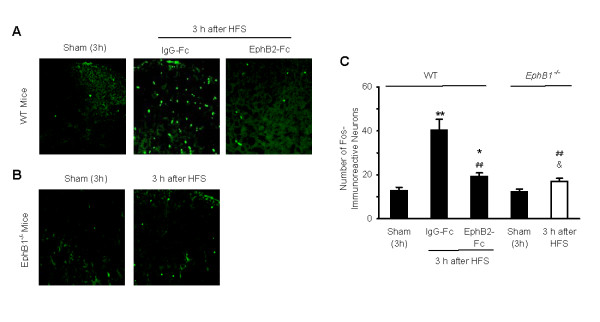
**Effects of EphB2-Fc and targeted mutation of EphB1 receptor on expression of c-Fos in the mouse DH following HFS *in vivo***. Examples of Fos-immunoreactive neurons are given in A (wild type mice, WT) and B (EphB1 receptor protein-null mice, *EphB*^-/-^) and data summarized in C. Five groups of mice (n = 5 each group) were tested and examined, the sham groups received no HFS (A and B) and the other groups received HFS with pretreatment of IgG-Fc or EphB2-Fc (A) HFS alone (B). * *p *< 0.05, ** *p *< 0.01 indicates significant differences compared with the WT-Sham. ## *p *< 0.01 indicate significant difference compared with WT-HFS-IgG-Fc (as control). &*p *< 0.05 indicate significant difference compared with *EphB1*^-/-^-Sham.

We further examined the effects of EphB2-Fc application and targeted mutation of the EphB1 receptor on HFS-induced phosphorylation of CaMKII, ERK and CREB proteins and c-Fos expression. The results showed that HFS-induced increases of p-CaMKII (10 min), p-ERK (10 min) and p-CREB (2 h) protein levels were partly, but significantly reduced by pretreatment of EphB2-Fc (2 μg, covering from 30 min prior to, during and up to 10 min to 2 h after HFS). As shown in Fig. 6A–D, approximately 40% of the increased p-CaMKII (Fig. 6A, B) and 70–80% of the increased p-ERK (Fig. 6A, B) and p-CREB (Fig. 6C, D) were inhibited, respectively. Further, targeted mutation of the EphB1 receptor completely blocked HFS-induced increases of p-CaMKII, p-ERK (Fig. 6E, F) and p-CREB (Fig. 6G, H). Similarly, HFS-induced c-Fos expression was significantly reduced by EphB2-Fc (2 μg, from 30 min prior to 3 h after HFS) in WT mice, or prevented by targeted mutation of the EphB1 receptor in the *EphB1*^-/- ^mice. Representative photomicrographs and the corresponding counts of Fos-like immunoreactive neurons in DH are shown in Fig. 7.

## Discussion

### An in vivo mouse model of LTP and its functional role in pain pathways

The primary field potentials recorded in this study have long latencies, high thresholds, long chronaxie, and a negative focus in the superficial laminae of DH. Such field potentials observed in the mouse DH is similar to that recorded in the rat [4,9] and in agreement with the conclusion by Schouenborg [28] that the late field potentials are generated primarily by synapses between C-afferent fibers and second-order neurons. The high-frequency, high-intensity tetanic stimulation to the ipsilateral sciatic nerve can induce a stable, long-lasting potentiation of the field potential in the mouse superficial DH, the LTP. The LTP can be suppressed by pre-treatment of MK-801, indicating that this LTP is an NMDA receptor-dependent synaptic potentiation. In addition, Ca^2+^-dependent pathways such as CaMKII and ERK are activated and phosphorylation of the transcription factor CREB is potentiated by HFS in the mouse SC. Therefore, the LTP we have recorded in the mouse DH *in vivo *has properties that are similar to LTP in the rat DH [3,4,9,14,15]. A recent study has demonstrated that the primary afferent C-fibers induce LTP of C-fiber-evoked excitatory postsynaptic currents (EPSCs) in projection neurons, but not in unidentified neurons in laminae I of SC slice of young rats [8], supporting the hypothesis that the potentiation of synapses between afferent C-fibers and projection neurons in the DH contributes to LTP.

LTP of synapses between primary afferent C-fibers and DH neurons has important functional roles in pain pathways [3]. Modulation of synaptic strength is a powerful mechanism to control signal flow through selected pathways. A typical consequence of LTP at excitatory synapses would be an increase in action potential firing of the same and perhaps also of downstream neurons in response to a given stimulus. The signal transduction pathways involved in LTP, including PKC, CaMKII, PKA, PLC, IP_3 _receptors, NOS and ERK [6,8,13-15,29] are also required for full expression of hyperalgesia in animal models of inflammatory and neuropathic pain [30-33]. In addition, high-frequency stimulation of the sciatic nerve fibers which induces LTP at synapses of C-fibers in the SC has behavioral consequences in rats and causes thermal hyperalgesia at the ipsilateral hind paw for approximately one week [6]. Thus, LTP at C-fiber synapses has a direct impact on nociceptive behavior.

Genetically modified mice, but not rats, have been widely used in investigations in many areas including pain for decades. This study provides an *in vivo *mouse model of LTP that should be very valuable for genetic approaches to the study of synaptic plasticity of the DH neurons.

### Potential mechanisms of the LTP

Although mechanisms of LTP at C-fibers synapses remain unclear, there is evidence for a postsynaptic, Ca^2+^-dependent form of LTP induction in lamina I neurons of the SC. Induction of LTP requires co-activation of neurokinin 1 (NK1) and neurokinin 2 (NK2) receptors [4], opening of T-type voltage-gated calcium channels [8,13], and activation of group I metabotropic glutamate receptors [34]. Activation of NK1 receptor by substance P (SP) may directly enhance single NMDA channel opening [35] and NMDA receptor mediated currents in lamina I neurons [13]. All of these may lead to substantial rise in postsynaptic [Ca^2+^]_i_, which is essential for LTP induction. In addition, Ca^2+ ^influx through Ca^2+^-permeable a-amino-3-ydroxy-5-methyl-4-isoxazolepropionic acid (AMPA) receptors may also be required for LTP induction in pain pathways [36,37]. Signal transduction of LTP involves Ca^2+^-dependent pathways including PKC, CaMKII, PKA, NOS and members of MAPK including ERK [6,8,13-15,29]. The potential mechanisms of LTP at synapses between C-fibers and SC projection neurons were recently reviewed by Sandkühler [3]. According to this model, LTP can be prevented if release of glutamate and/or SP is inhibited, or if opening of voltage sensitive and Ca^2+ ^permeable ion channels is blocked. LTP deficits or de-potentiation could result from de-phosphorylation of synaptic proteins, changes in receptor trafficking or degradation of synaptic proteins [3]. Studies have shown that in the rat DH this synaptic plasticity is sensitive to inhibitors of iNOS, glial cell metabolism [38], group I mGluR [34], glial glutamate transporters [39,40] and protein synthesis [41].

This study presents the first description of the time course of HFS-induced phosphorylation of CaMKII, ERK and CREB and altered expression of c-Fos in the SC of mice and rats. Note that the transient, peak phosphorylation of both CaMKII and ERK occured within 5–10 min of HFS, while phosphorylation of CREB reached its peak approximately 2–3 h after HFS. Increased expression of c-Fos was first increased at 2 h and reached peak at 3 h after HFS. Our results also showed obvious differences in the HFS-induced c-Fos expression in mice and rats, 1) the peak expression of c-Fos in the mouse DH was detected at 3 h, whereas it was markedly increased in the DH of rats at 2 h [40]; and 2) the Fos-immunoreactive neurons were seen distributed across all of the mouse DH, while it distributed mainly in the superficial layers of rats DH [40] (and the present analysis, data not shown).

### EphB1 receptor, a mechanism underlying LTP

The Eph receptors, constituting the largest group of RTKs in mammals, with 13 members divided into an A subclass (EphA1–EphA8) and a B subclass (EphB1–EphB4, EphB6) [42,43], have been indicated to play important roles in synaptic plasticity in the nervous system [16-24,26,44,45]. We have recently found evidence that the EphB receptors play an important role in development of LTP in the DH of rats [9]. Using our new *in vivo *mouse model of LTP described here and taking advantage of EphB1 receptor protein-null mice, this study demonstrates that the EphB1 receptor is essential to the development of LTP. Targeted mutation of the EphB1 gene successfully prevented development of LTP and suppressed the associated activation (phosphorylation) of CaMKII, ERK, and CREB as well as increased c-Fos expression.

The ephrinB-EphB interaction is well known to mediate bidirectional signals that propagate into EphB-expressing cells (forward signaling) and ephrinB-expression cells (reverse signaling). Both forward signaling and reverse signaling can play important roles in synaptic plasticity, acting either pre- or postsynaptically. Recent studies have proposed mechanisms for potential interactions between ephrinB-EphB receptors and AMPA, NMDA and mGlu receptors [46-49]. EphB receptors interact with numerous PSD-95, Discs-large Zoe-1 (PDZ) domain that can regulate the membrane trafficking of AMPA receptor subunits, such as PSD-95 itself, the glutamate receptor-interacting protein (GRIP) and protein interacting with C kinase-1 (PICK-1). Furthermore, phosphorylation of synaptojanin by EphB2 receptors modulates clathrin-mediated endocytosis of the GluR1 subunit of AMPA receptors [49]. Interaction between EphB receptors and GRIP or PICK-1 may instead affect membrane trafficking of the GluR2 subunit of AMPA receptors [48]. Both ephrins and EphB receptors have been found to be co-localized with NMDA receptors post-synaptically and to positively modulate NMDA receptors through a physical interaction between the N-terminal domains, or through the intervention of signaling molecules such as Src or other intracellular proteins. The activation of ephrinBs amplifies group-1 mGlu receptor signaling through mechanisms that involve NMDA receptors and/or PDZ/regulator of G-protein signaling (RGS) [46,47]. The interaction with ionotropic or metabotropic glutamate receptors provides a substrate for the emerging role of ephrins and Eph receptors in the regulation of activity-dependent forms of synaptic plasticity.

It has been reported that a selective impairment of long-lasting LTP induced by beta-burst stimulation of Schaffer collaterals [18] and both early and late phases of LTP induced by HFS were attenuated [19] in EphB2 receptor protein-null mice. Our results here show that targeted mutation of EphB1 receptor can prevent development of LTP and the associated phosphorylation of CaMKII and ERK and CREB and expression of c-Fos in the mouse SC. We hypothesize that this mechanism of ephrinB-EphB receptor signaling, particularly the EphB1 receptor, may apply to LTP at synapses between the primary afferent C-fibers and DH neurons of mice and rats. In addition, taken together with the present and our earlier findings that nerve injury fails to produce neuropathic thermal hyperalgesia in *EphB1*^-/- ^mice [50], we have growing evidence supporting the hypothesis that the LTP in the DH may contribute to hyperalgesia.

## Conclusion

The present study describes an *in vivo *mouse model of LTP at synapses between primary afferent C-fibers and superficial DH neurons that is useful for probing pain mechanisms, including the DH neuron excitability and their synaptic plasticity and hyperalgesia in genetically altered mice. Further we have shown that the EphB1 receptor is critical to development of LTP and activation of CaMKII and ERK pathways, and the subsequent activation of CREB and induction of c-Fos expression.

## Methods

### Animals

The generation of EphB1 receptor protein-null mutant mice has been previously described [51,52]. For the present study *EphB1*^+/- ^heterozygous males and females in the CD1 background were bred to obtain a cohort of homozygous knockout (*EphB1*^-/-^) and wild-type (WT) (*EphB1*^+/+^) control littermate adult mice (25–30 g-wt). All breeding was done by the Henkemeyer group and adult mice were provided to the Song group, who were blinded to the genotypes. Additional WT male CD1 mice (25–30 g-wt) were obtained from Charles River Laboratories (Wilmington, MA). All experimental procedures were conducted in accordance with the regulations of the ethics committee of the International Association for the Study of Pain and approved by the University of Texas Southwestern Medical Center and Parker Research Institute Animal Care and Use Committee, respectively.

### In vivo Extracellular recordings of LTP in mice DH

Protocols for mice preparation and for *in vivo *extracellular recordings of the C-fiber-induced field potential and LTP were modified from those we have recently described in rats [9]. Urethane (1.5 g/kg, given intraperitoneally, i.p.) was used to induce and maintain anesthesia. All surgeries and the *in vivo *electrophysiological recordings were performed under anesthesia. The trachea was cannulated to allow mechanical ventilation with room temperature air, if necessary. Colorectal temperature was monitored and kept at ~37–38°C via a feedback-controlled heating pad under the ventral surface of the abdomen. In each experiment phosphate-buffered saline was injected (i.p., 2 ml prior to surgery and 1 ml every 2 h) to provide fluid and maintain electrolyte balance. A laminectomy was performed to expose the lumbar enlargement of the SC at L_5_-L_6 _for electrophysiological recording and the left sciatic nerve was prepared for stimulation. The mouse was stabilized on the stereotaxic frame, the exposed cord was covered with warm agar (2% in saline) and the sciatic nerve covered with paraffin oil. After the agar hardened, a small hole was made above the recording site for application of drug or vehicle.

Field potentials evoked by peripheral stimulation of C fibers were recorded at a depth of 100–500 μm (Fig. 1A and 1B) and 150–300 μm (Fig. 1C and D and all of the other recordings) from dorsal surface of the SC with glass capillary microelectrodes (DC resistances 3–5 MΩ filled with 2 M NaCl), which were driven by an electronically controlled microstepping motor (MHW-3, Narishinge, Japan). The depth of the electrode within the DH was judged by the microdrive readings. In order to locate the recording site, some electrodes were filled with fast blue and the dye was ejected at the end of experiments. The SC segment was then removed, sectioned and examined under microscope (Fig. 1E–G). Axoclamp 2B and DigiData 1200 amplifiers and PCLAMP-8 (Axon Instruments, Foster City, CA) were used for data acquisition and analysis. The signals were filtered (bandwidth: 0.1–500 Hz) and recorded at a sampling rate of 10 kHz. The field potentials were recorded at 1-min intervals and then 5 recordings were averaged and presented as 1 datum point in the results. The sciatic nerve was stimulated by a bipolar platinum hook electrode. Single square pulses (0.5 ms duration) were delivered once a minute to the sciatic nerve and used as test stimuli. The strength of stimulation was adjusted to 2.5 times the threshold for a C-fiber response. The LTP was induced by HFS consisting of 100 electrical pulses, each 0.5 ms, 100 Hz, at 5× the threshold current of C-fibers, given in 4 trains of 1-s duration at 10-s intervals to the sciatic nerve. At the end of each experiment, the animals were sacrificed by an overdose of urethane (6 g/kg, i.p.).

### Immunoblot analysis

Immunoblot analysis was used to detect p-CaMKII, p-ERK and p-CREB protein levels in the SC. Lumbar segment of the SC from one mouse were pooled for each sample and each group consisted of four samples. The procedure used to quantify temporal changes in protein levels was similar to that previously described [27,53]. Whole cell protein extracts lysates were used. After transfer to nitrocellulose filters, the filters were blocked with 2% bovine serum albumin (BSA) and then incubated overnight at 4°C with the primary antibodies [p-CaMKII (Thr286) 1:1000; p-ERK1/2 (Thr202/Tyr204) 1:500 from Cell Signaling Technology, Danvers, MA; p-CREB (Ser133) from the Santa Cruz; CA; GAPDH 1:1000 from Sigma, St Louis, MO]. The filters were developed using ECL reagents (Perkinelmer, MA) with secondary antibodies from R&D (Minneapolis, MN). Data were analyzed with the Molecular Imager (ChemiDoc XRS) and the associated software Quantity One-4.6.5 (Bio-Rad Laboratories, Hercules, CA).

### Immunohistochemical and immunofluorescence staining of c-Fos

The immunofluorescence staining was performed as we have recently described [27,54]. Briefly, the lumbar segment of the SC was dissected out of mice, post-fixed, and then the embedded blocks were sectioned (10 μm thick). Sections from each group (4–5 mice in each group) were incubated with rabbit anti-c-Fos polyclonal antibody (1:100), (Santa Cruz Biotechnology Inc. Santa Cruz, California, USA). Rabbit IgG (1:200, Vector Laboratories, Inc., Burlingame, California, USA) was used as an isotype control. The morphologic details of the immunofluorescence staining on the SC were studied under a fluorescence microscope (Olympus BX51WI; Olympus America Inc., Melville, New York, USA). Images were randomly coded and transferred to a computer for further analysis. Fos-immunoreactive neurons were counted in blind fashion. The number of Fos-like immunoreactive neurons in DH was determined by averaging the counts made in 20 sections (L_4_-L_5_) for each group.

### Drug application

Contributions of EphB receptor signaling to the LTP was examined partly by applying an EphB receptor blocking reagent onto the SC in addition to using the EphB1^-/- ^mice. The EphB receptor blocking reagent EphB2-Fc chimera (2 μg, Sigma, St Luis, Missouri, USA), the human IgG-Fc fragment (Fc control, 2 μg) (Jackson Laboratory, Bar Harbor, Maine, USA), and an NMDA receptor antagonist MK-801 (5 μg) (RBI, Natick, MA) were administrated topically to the small hole previously made in the agar above the recording site of the SC.

### Statistical tests

SPSS Rel 15 (SPSS Inc., Chicago, Illinois, USA) was used to conduct all the statistical analyses. The nonparametric Wilcoxon signed-rank test and the Kruskal-Wallis test were used to test the field potential and LTP of DH neurons within and between the groups, respectively. Alteration of phosphorylation of the protein levels and expression of the c-Fos detected were tested with one-way ANOVA, with repeated measures followed by Bonferroni post hoc tests. All data are presented as mean ± SE. Statistical results are considered significant if *p *< 0.05.

## Abbreviations

AMPA: a-amino-3-hydroxy-5-methyl-4-isoxazolepropionic acid; ANOVA: analysis of variance; CaMKII: Ca^2+^/calmodulin-dependent kinase II; CREB: cyclic AMP related element binding; DH: spinal dorsal horn; EPSC: excitatory postsynaptic current; ERK: extracellular signal-regulated kinase; GRIP: glutamate receptor-interacting protein; HFS: high frequency stimulation; LTP: long-term potentiation; MAPK: mitogen activated protein kinase; NK1: neurokinin 1; NK2: neurokinin 2; NMDA: N-methyl-D-aspartate; NO: nitric oxide; NOS: nitric oxide synthase; PDZ: Discs-large Zoe-1; PICK-1: protein interacting with C kinase-1; PKC: protein kinase C; PKA: protein kinase A; RGS: regulation of G-protein signaling; RTK: receptor tyrosine kinase; SC: spinal cord; SP: substance P; WT: wild-type.

## Competing interests

The authors declare that they have no competing interests.

## Authors' contributions

WTL and YH conducted the electrophysiological and immunoblot studies and data analysis. HCL and WTL conducted the immunohistochemical and immunofluorescence staining of c-Fos and data analysis. BA and JHZ involved in immunoblot and electrophysiological studies, respectively. YPW involved in planning the project. MH generated and identified the EphB1 receptor protein-null mutant mice and kept stimulating discussion on the project. XJS developed and supervised the project, designed the experiments, conducted some data analysis and prepared the manuscript. All the authors approved the final manuscript.
